# Supplementary material for: Maternal age affects equine day 8 embryo gene expression both in trophoblast and inner cell mass
Source: BMC Genomics. 2022 Jun 15;23:443. doi: 10.1186/s12864-022-08593-7 (PMC9199136; doi:10.1186/s12864-022-08593-7)
Supplement: Supplementary file 1 — Additional file 1: Supplementary Figure 1. Analysis of ICM enriched and TE part before gene expression deconvolution using DeMixT. On first page, the analysis of differential expressed genes is represented. The second page represent the results of the gene set enrichment analysis of ICMandTE gene expression. The third page presents the clustering of gene sets altered by maternal age from SUMER analysis of the GO BP and KEGG terms of mixed ICMandTE and TE_part. [file 12864_2022_8593_MOESM1_ESM.pdf]

A

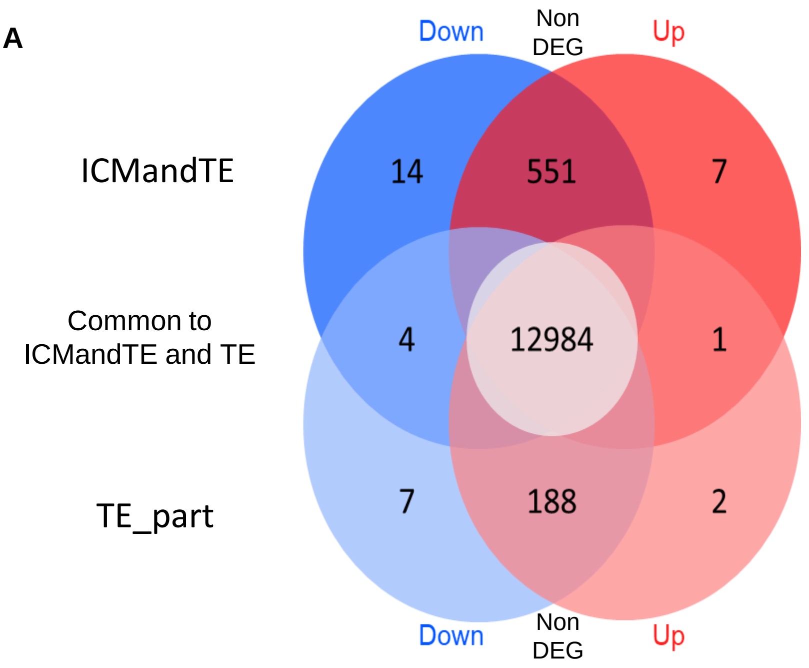

B

	ICM and TE	pure TE
CST6	0.96	0.87
ENSECAG00000035582	0.8	0.75
GALM	0.66	0.61
GTPBP8	0.69	0.58
GNG10	0.42	0.37
NMI	2.28	0.35
MGST1	0.47	0.35
USP11	-0.53	-0.15
PARP1	-0.56	-0.43
DESI1	-0.57	-0.3
MARS2	-0.59	-0.14
MKI67	-0.59	-0.42
MYH10	-0.62	-0.35
PCM1	-0.64	-0.28
PPFIBP1	-0.72	-0.05
H1-4	-0.79	-0.87
PHKA2	-1.06	-0.6
KMT2A	-1.1	-0.17
MAP1B	-1.41	0.42
DPYSL5	-2.12	-0.53
PAK3	-3.91	

C

	ICM and TE	pure TE
CD69		5.09
PFAS	-0.34	-0.38
CBS	-0.49	-0.37
SRGAP3	-0.55	2.52
SETX	-0.56	-0.49
LUZP1	-0.72	-0.75
REV3L	-0.37	-0.75
ATAD2B	-0.52	-0.93
ENSECAG00000022280	-1.7	-2.53

D

	ICM and TE	pure TE
RPS29	0.95	0.78
AASS	-0.89	-0.7
H4C3	-1.15	-1.33
RNASE1	-2.06	-2.14
ENSECAG00000038654	-1.96	-2.48

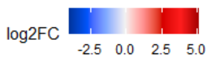

<u>**Venn diagram (A) and tables of DEG in TE_part and ICMandTE according to maternal age (B, C and D)**</u>

Table B presents DEGs in ICMandTE, Table C presents DEGS in TE_part and Table D presents DEGS in common in both parts. Blue represents down regulated DEG (log2FC<0) while red show up regulated DEG (log2FC>0) in OM embryos compared to YM embryos. DEGs are in Entrez Gene ID or Ensembl Equine Gene ID (ENSECAG) when Entrez Gene ID was not available.

DEG: Differentially Expressed Genes (padj<0.05); log2FC: Fold change in log2; TE: Trophoblast; ICM: Inner Cell Mass; OM: group of embryos produced by Old Multiparous mares; YM: group of embryos produced by Young Multiparous mares

ICMandTE

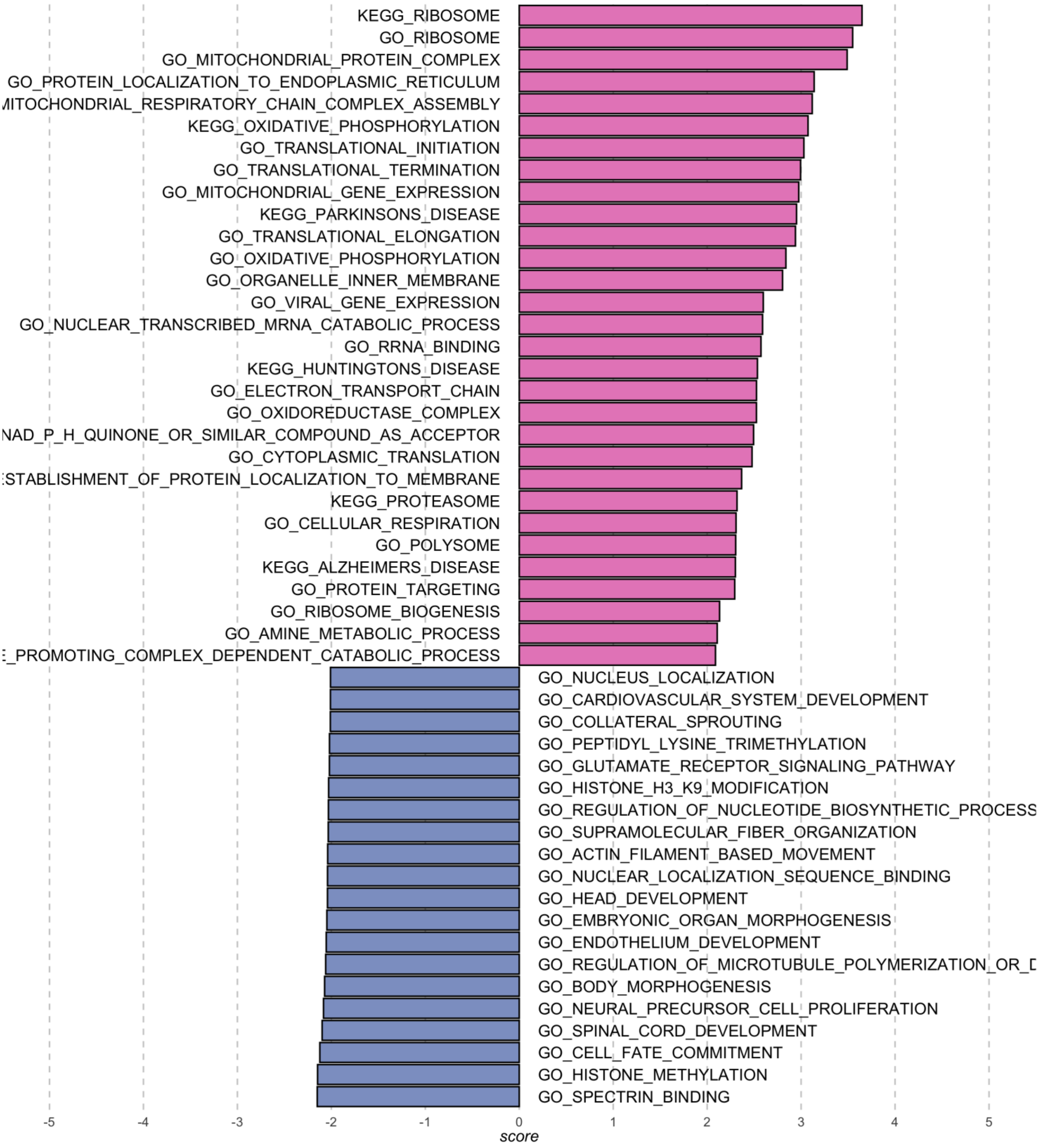

Diagram of most perturbed GO and KEGG pathways (FDR < 0.05) in inner cell mass enriched part (ICMandTE) of equine D8 blastocysts according to maternal age

Pink pathways are the most enriched pathways in embryos from old multiparous mares and purple pathways are the most enriched pathways in embryos from young multiparous mares. Score represents False Discovery Rate using a Gene Set Enrichment Analysis.

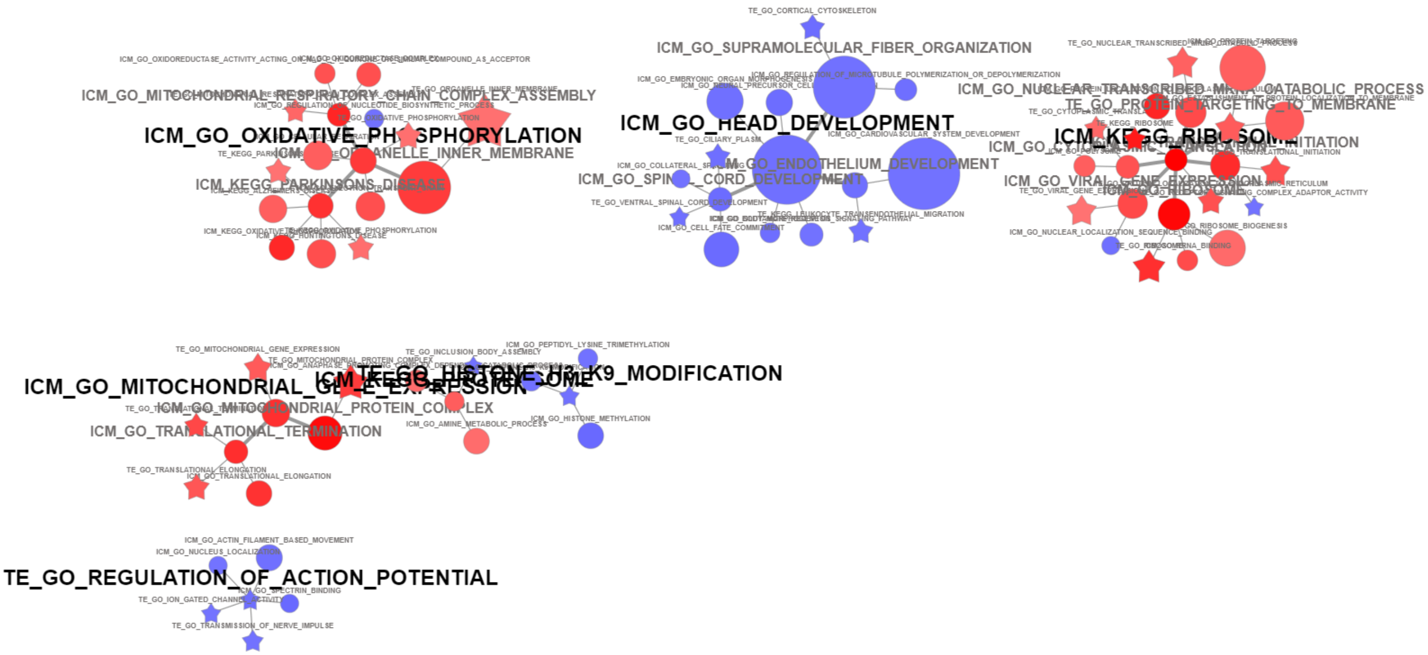

<u>Clustering of the most perturbed GO and KEGG terms in ICMandTE and TE according to mares' age</u>

Graph representing in nodes GO Biological process and KEGG representative terms in ICMandTE (circle) and TE_part (star). Node size represents the size of the gene set. Blue nodes represented gene sets enriched in YM (NES < 0) while red nodes represented gene sets enriched in OM (NES > 0). Edges represent the level of connection between representative gene sets. This graph was performed using SUMER R package [55].

NES: Normalized Enrichment Score; ICM: Inner Cell Mass; OM: group of embryos produced by Old Multiparous mares; YM: group of embryos produced by Young Multiparous mares
