# Supplementary figures and images for: Maternal age affects equine day 8 embryo gene expression both in trophoblast and inner cell mass
Source: BMC Genomics. 2022 Jun 15;23:443. doi: 10.1186/s12864-022-08593-7 (PMC9199136; doi:10.1186/s12864-022-08593-7)

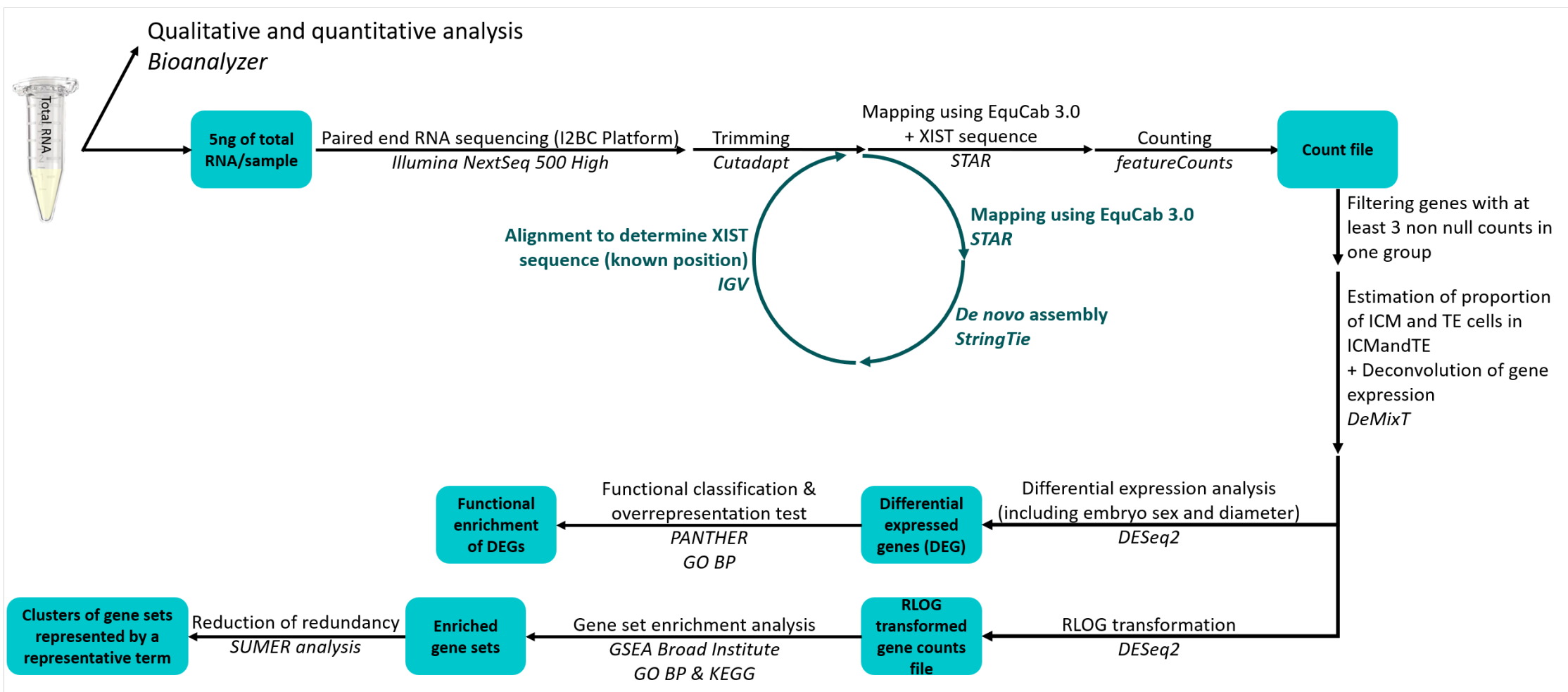

Supplement: Supplementary file 2 — Additional file 2: Supplementary Figure 2. Pipeline of biostatistical analysis. Once extracted, total RNA qualification and quantification were obtained using a Bioanalyzer. For sequencing, 5 ng/sample of total RNA were used for paired end RNA sequencing (I2BC platform) using Illumina NextSeq 500 high technology. Sequences were trimmed using Cutadapt. To determine XIST sequence, a de novo assembly was made using a mapping with STAR and StringTie. The GTF observed was aligned in IGV and XIST sequence was found at the known position. XIST sequence was included in EquCab 3.0 and a new mapping was performed using STAR. Counting was performed using featureCounts. The differential analysis was performed including embryo sex and diameter using Deseq2. A functional enrichment analysis of DEGs was performed using PANTHER web software. In another time, counts of all genes were normalized using Deseq2 and a gene set analysis (GSEA) was performed using the Gene Ontology (GO) biological process (BP) and the Kyoto Encyclopedia of Genes and Genomes (KEGG) databases with GSEA software form the Broad Institute. To reduce redundancy between terms, SUMER analysis was performed on enriched gene sets. [file 12864_2022_8593_MOESM2_ESM.pdf]
